# AI-enabled predictive, preventive and personalised oral health management: a lightweight patient-centred model for automated assessment of dental plaque and gingival inflammation

**DOI:** 10.1007/s13167-025-00432-5

**Published:** 2026-02-24

**Authors:** Camila Lindoni Azevedo, Ryan Banks, Vishal Thengane, Teresa Cristina Alves da Silva Gonzalez Carvalho, Fausto Medeiros Mendes, Yunpeng Li, Edgard Michel Crosato

**Affiliations:** 1 Present Address: Faculty of Dentistry, University of São Paulo, São Paulo, Brazil; 2 School of Computer Science and Electronic Engineering, University of Surrey, Guildford, UK; 3 Faculty of Dentistry, Oral & Craniofacial Sciences, King’s College London, London, UK

**Keywords:** Oral health, Preventive care, Artificial intelligence (AI), Dental plaque, Gingivitis, Deep learning, Periodontal disease, Chronic inflammation, Predictive diagnostics, Predictive preventive personalised medicine (PPPM), Digital health self-monitoring, Personalised maintenance, Patient phenotyping, Digital biomarkers

## Abstract

**Rationale:**

Periodontal diseases are highly prevalent and largely preventable. The challenge of ensuring sustained adherence to preventive measures, such as mechanical plaque control, remains unresolved despite their strong scientific support. Embedding emerging strategies within the framework of predictive, preventive, and personalised medicine (PPPM) offers a promising path to improve adherence, enable early risk prediction, and tailor interventions. Within this paradigm, digital image biomarkers are increasingly recognised as essential tools for supporting proactive, system-oriented oral health management.

**Working hypothesis and methodology:**

This study hypothesised that a patient-centered artificial intelligence (AI) model could automatically detect dental plaque and gingival inflammation, advancing predictive and preventive strategies for oral health. To verify the working hypothesis, a calibrated periodontist annotated 504 intraoral images, generating target masks (TM) as ground truth. A YOLOv8Seg-based deep learning model was trained for simultaneously segmented teeth, dental plaque, and gingival health status (healthy vs. inflamed), generating predicted masks (PM) that were subsequently used for classification tasks, including the calculation of gingival and plaque indices.

**Results:**

The model achieved moderate segmentation performance (IoU = 47%, DSC = 61%), with higher accuracy for tooth regions (mAP = 71% and 77%). Detection of dental plaque, healthy gingiva, and inflamed gingiva showed moderate precision (52%). Plaque index classification performed strongly (DSC = 95%, recall = 91%), whereas gingival inflammation showed moderate but clinically meaningful accuracy (DSC = 70%, recall = 92%), supporting early identification of inflammatory burden.

**Conclusions:**

The proposed model functions as a practical digital tool for predictive oral healthcare by generating actionable, image-based biomarkers for patient phenotyping, early risk flagging, and site-specific behavioural reinforcement. Its lightweight architecture enables future integration into mobile platforms for longitudinal digital health monitoring and precision prevention. Expert recommendations include embedding AI-based plaque and gingivitis assessment into mobile health tools to enhance participatory self-monitoring; integrating imaging-derived biomarkers with behavioural, microbiological and socioeconomic information to support multimodal diagnostics and more accurate patient profiling; operationalising targeted prevention through site-specific alerts and personalised recall strategies; and deploying lightweight AI solutions in community-level screening programmes to reduce the burden of chronic inflammatory oral conditions. This work supports the transition from reactive treatment to a proactive, system-oriented PPPM.

**Supplementary Information:**

The online version contains supplementary material available at 10.1007/s13167-025-00432-5.

## Introduction

Dental biofilm is the primary etiological factor for the most prevalent oral diseases, including tooth decay and periodontal disease [1]. The interactions among the microorganisms in the biofilms can increase the risk of disease leading to a cariogenic and/or a periodontal challenge which, if uncontrolled, may lead to the destruction of dental and periodontal structures and eventual tooth loss [2]. These pathological processes are often accompanied by visible clinical signs, such as the accumulation of dental plaque and gingival inflammation presence [3].

Embedding preventive approaches within a predictive, preventive, personalised and participatory (5Ps) framework underscores the need for innovative, patient‑centred strategies that not only target disease but also empower individuals to manage their own oral health more effectively. Predictive, Preventive, and Personalised Medicine (PPPM) is an innovative healthcare paradigm that shifts the focus from reactive treatment of established disease to early risk assessment, targeted prevention, and tailored interventions. Integrating oral health into the broader PPPM/5Ps paradigm promotes early risk assessment, targeted prevention, and personalised interventions that can significantly improve long-term outcomes [4, 5].

The principal goal for oral health care is to achieve a high standard plaque control through patient self-care [6]. This involves the regular removal of dental biofilms using oral hygiene devices such as toothbrush, floss, interdental sticks, interdental brushes, and oral irrigators. While daily toothbrushing with fluoride toothpaste is universally recommended, consistent and effective plaque control is rarely achieved in the general population [7].

There is a great opportunity for self-monitoring oral health through automatic analysis of intraoral images, supporting participatory and prevention-oriented strategies highlighted in contemporary oral healthcare frameworks [8]. Smiling intraoral images can help detect the presence of dental plaque and the relation between plaque adhesion area and teeth area provides reliable information about plaque index. Our research group recently developed the selfie dental plaque index, a quantitative method to assess dental biofilm that provides a permanent record that can be reassessed later and create an image comparison repository [9]. Intraoral images acquisition is applicable with many types of cameras, including smartphones, and can be incorporated on a daily basis routine for self-monitoring oral hygiene quality [10].

Advances in image-based diagnosis using artificial intelligence (AI) offers possibilities in the field of medicine and dentistry. AI refers to the capability of machines that exhibits a form of its own intelligence, learned through previous input data. Deep learning (DL) is a component of machine learning that utilizes the network with different computational layers in a deep neural network to analyze the input data. AI has been used in dentistry to improve the process of diagnosis and quality patient care. Studies have used tomographic images, lateral cephalometric radiographs, bitewing radiographs, facial photos, panoramic radiographs [11].

Recent research has demonstrated the feasibility using AI for detecting dental biofilm in digital images, potentially enhancing oral hygiene [12–14]. Regarding gingivitis detection, studies have shown that AI can successfully distinguish gingivitis and healthy through intraoral images [15–18]. The overall mean rate of these AI models achieves 70% of sensitivity, sensibility, accuracy, and precision. Despite that, these studies analysed a low-variability dataset [11–13, 18] or image inclusion criteria were difficult to apply in health promotion [14, 16]. Additionally, some studies did not provide detailed information on the architecture of the AI, making reproducibility impossible [15]. Only one study attempted to evaluate gingivitis and the presence of plaque simultaneously, but the examiners did not label the plaque area [17].

This study aimed to develop a patient-centered DL-based automated tool to detect dental plaque, signs of gingival inflammation using a varied dataset of intraoral images. To the best of our knowledge this is the first study that aims to evaluate the performance of an AI-driven model for periodontal clinical indexes, commonly used in the primary prevention of periodontal disease. The outcomes include teeth area, dental plaque area, dental plaque index, gingival status (gingivitis or healthy) and gingival index. Yolov8-Seg is a single-shot detector model regarded as the new state-of-the-art, optimizing both speed and accuracy [19]. The development of an algorithm for automatic detection of dental plaque and gingival inflammation could lay the foundation for future tools to improve oral hygiene quality, self-awareness, and health promotion.

### Working hypothesis

A lightweight, patient-centred AI model for intraoral image analysis could advance the three pillars of PPPM. We hypothesised that the model would (1) enable predictive diagnostics by flagging early indicators of plaque accumulation and gingival inflammation; (2) provide site-specific feedback to guide improved self-care and reduce risk of disease progression; and (3) offer pragmatic personalisation through longitudinal self-monitoring and rule-based notifications prompting timely self-care and referral.

## Materials and methods

The study was described according to the Checklist for AI in Dental Research [20].

### Dataset

The intraoral images were randomly selected between September 2021 and December 2023 from a radiology private clinic (Ethical approval 5.681.738). These images were obtained from pre-orthodontic treatment patients with no exclusion criteria related to image quality or clinical condition (e.g. dental caries, periodontal disease, orthodontic appliances).

The sample comprised 168 subjects and 504 images, with each patient contributing three standardized intraoral photographs (buccal left-side view, buccal right-side view, and buccal frontal view). The sample exhibited meaningful demographic diversity, with a mean age of 31.5 years (range 8–75 years), 56% female and 44% male, and 5% (27 images) had fixed orthodontic appliances. All images were anonymized, and each subject was represented by a randomized identification code.

### Examiner reliability and annotation workflow

The reference annotations (target mask) were collected from a calibrated periodontist. The examiner’s reliability had been previously validated in a clinical–photographic study [9], demonstrating strong intra-examiner consistency when assessing plaque distribution across different tooth regions (*r* = 0.8). Additionally, the study reported good consistency between clinical plaque assessment and the plaque area measured from intraoral images (*r* = 0.6).

**Fig. 1:**
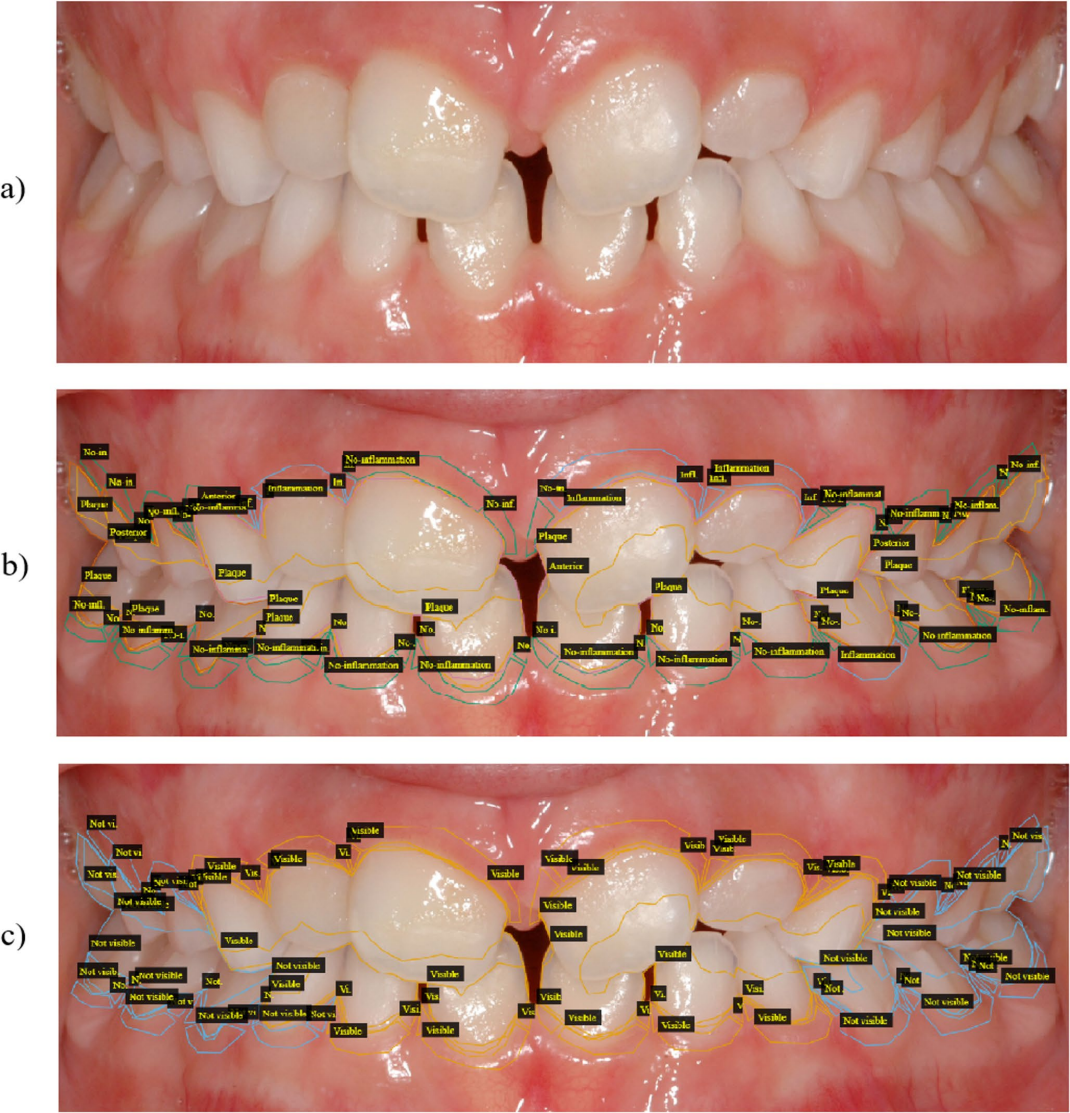
Annotation workflow. **a** intraoral original image, **b** class instances, and (**c**) classification-eligible regions

Each dataset image was labelled using the annotation open-source software VGG Image Annotator [21]. First, using the polygon tool shape, the tooth area with plaque presence was annotated. Changes in tooth colour, brightness and texture were considered indicative of dental biofilm. Besides, mineralized bacterial plaque (dental calculus) was labelled as plaque area. Anterior tooth area was determined using the polygon shape and included incisive teeth and canines. Posterior teeth area was selected with polygon shape and was represented by premolars and molars. Three gingival sites per tooth (mesiobuccal, buccal and distobuccal) were evaluated. Signs of gum inflammation included changes in colour and texture of the soft marginal gingival tissue. Absence or presence of inflammation on each tooth surface was recorded in a dichotomous manner (healthy or gingivitis) resulting in a percentage of sites with gingivitis signs (Fig. 1b).

On frontal images, anterior teeth and the respective gingival sites, are visible and were annotated as “classifiable area”, while posterior areas that were not fully visible were annotated as “non-classifiable”. On lateral images (left and right side) anterior teeth and gingival sites were annotated as “non-classifiable”, and posterior teeth and gingiva are visible and were annotated as “classifiable area” (Fig. 1c). Plaque area, anterior teeth area, posterior teeth area, healthy gingival site and inflamed site were classified as visible or not visible, depending on image type (Suppl. S1).

The images in the dataset may contain complete or incomplete teeth, all visible tooth areas were selected and identified with the corresponding label. To further document dataset variability and class representation, all annotated instances per semantic category used in the model (anterior teeth, posterior teeth, dental plaque, healthy gingiva, inflamed gingiva, and background) were quantified (Suppl. S2).

###  Model

**Fig. 2:**
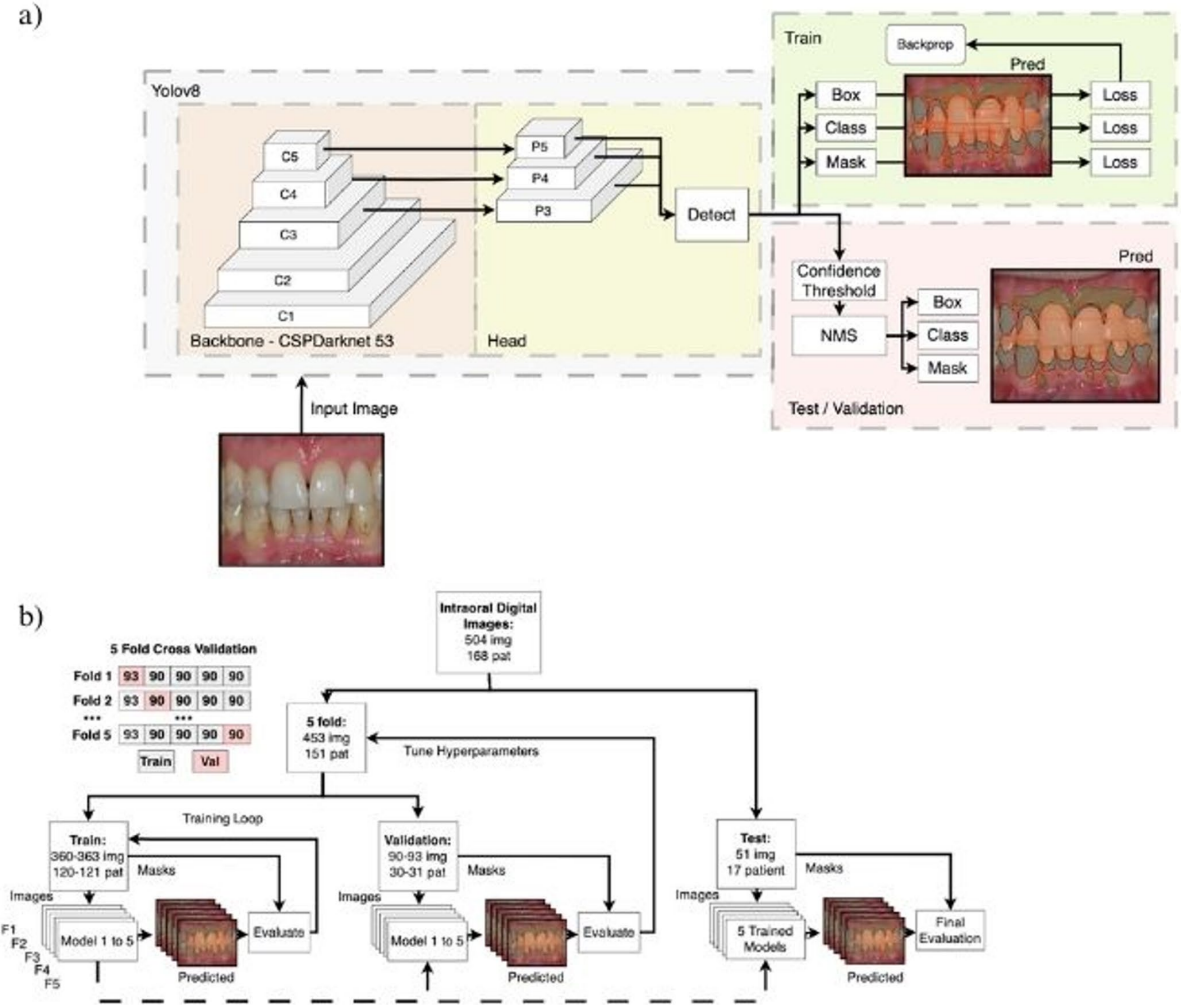
Pipeline of the AI-Model. **a** The simplified architecture of the YOLOv8seg model for intraoral image segmentation. **b** The 5-fold cross validation process

YOLO (You Only Look Once) framework is characterised as a state-of-the-art one-stage detection model. YOLO can be used as an instance segmentation model for automatic object detection and image segmentation. In the medical field, YOLO has been employed for cancer detection and skin segmentation [22]. In this study, a network structure based on the YOLOv8Seg architecture was employed for dental plaque and gingival inflammation detection. The input data were resized to 640 × 640 RGB colour images and the target masks (TM), which were created based on teeth area, plaque area and gingival status per site (Fig. 2a).

The dataset was first divided into a patient-wise split to prevent snooping bias, ensuring that all three images obtained from each patient remained in the same subset. A 10% hold-out test set (51 images; 17 patients) was reserved exclusively for final performance evaluation, with no exposure during model development. The remaining 90% (453 images) composed the training/validation pool. This subset was further partitioned using an alternating five-fold cross-validation scheme (Fig. 2b), where each fold contained approximately 20% validation (90–93 images) and 80% training (360–363 images). For each of the five iterations, one fold served as the validation set while the remaining four folds were used for training, resulting in five non-overlapping validation sets. Hyperparameters were tuned using grid-search optimisation integrated within the cross-validation loop. Average performance across the five folds was used as the final internal metric. This strategy maximises methodological transparency, reduces overfitting risk, mitigates data snooping bias, and enables a rigorous evaluation of model stability across unseen data [23].

###  Evaluation metrics

To quantitatively evaluate the performance of the proposed method, instance segmentation model performance and classification performance were evaluated against the target test data.

Model performance was evaluated by comparing the predicted test outputs against the TM annotations. For teeth evaluation, the statistical unit was pixel-wise (plaque/tooth) whereas for gingival evaluation, it was the site (inflamed/healthy). The evaluation metrics were pixel-wise mask Intersection over Union (IoU), and threshold object-wise mask precision, recall, Dice Similarity Coefficient (DSC) and mean average precision (mAP) [24]. The IoU threshold for Non-Maximum Suppression (NMS) and confidence threshold are 0.35 and 0.15, respectively, following standard practices in hyperparameter tuning for object detection models [22].

The evaluation metrics for classification performance were the average precision, recall, and DSC. The plaque index was determined by the percentage of the classifiable tooth area with plaque presence. We only considered the predicted plaque areas that overlap with the target classifiable tooth area. For gingival classification, two main criteria were used: (1) gingival site status and, (2) gingival site location. For the gingival site status, a threshold of 30% was considered. The predicted areas with gingival inflammation signs overlapping more than 30% (IoU ≥ 0.3) of a gingival site, were classified as inflamed gingiva. Less than 30% of overlap was considered healthy gingiva. Site location was determined by the Euclidean distance from target gingival site’s centroid to the closest pixel for both the classifiable and non-classifiable tooth area. Gingival sites located closer to non-classifiable tooth area were excluded from the evaluation. The gingival index was calculated as the percentage of predicted classifiable gingival sites showing inflammation relative to the total number of classifiable gingival sites. The cut-off point for both the plaque index and gingival index was 20% [25, 26].

##  Results

###  Quantitative analysis

Table 1 lists the quantitative comparison results for overall YOLOv8Seg model performance. The average DSC, precision and recall was 61% (0.609 ± 0.006, 0.613 ± 0.016, 0.608 ± 0.015, respectively) and the average IoU was 47% (0.471 ± 0.163). The mAP of YOLOv8Seg in detecting regions of interest was 0.603, for anterior teeth was up to 0.715, whereas for posterior teeth it was 0.766. The model’s capacity to distinguish healthy and inflamed gingiva was also moderate, healthy gingiva DSC was 54% (0.544 ± 0.008) and gingivitis (inflamed) DSC was 49% (0.487 ± 0.020).

**Table 1:** Quantitative results of tooth, plaque and gingival segmentation performance of YOLOv8seg model on the test dataset (mean and standard deviation)

Metric	IoU	Precision	Recall	DSC	mAP 0.5	mAP 0.5-0.95
Class						
Average	0.471 ±0.163	0.613 ±0.016	0.608 ±0.015	0.609 ±0.006	0.603 ±0.010	0.228 ±0.004
Anterior	0.602 ±0.068	0.712 ±0.033	0.684 ±0.030	0.698 ±0.030	0.715 ±0.029	0.263 ±0.019
Posterior	0.599 ±0.060	0.783 ±0.035	0.785 ±0.037	0.784 ±0.036	0.766 ±0.041	0.225 ±0.008
Plaque	0.528 ±0.071	0.523 ±0.039	0.540 ±0.016	0.531 ±0.020	0.545 ±0.018	0.227 ±0.006
Healthy Gingiva	0.411 ±0.008	0.526 ±0.025	0.567 ±0.029	0.544 ±0.008	0.513 ±0.011	0.212 ±0.006
Inflamed Gingiva	0.215 ±0.066	0.522 ±0.041	0.465 ±0.070	0.487 ±0.020	0.474 ±0.011	0.213 ±0.004

The obtained overall performance of the plaque segmentation showed moderate performance, the model predicted 53% of dental plaque (IoU = 0.528 ± 0.071). Despite that, the plaque classification performance, represented by plaque index, achieved 95% DSC, 91% recall and 98% precision (Table 2). For gingival segmentation and classification performance however, the model was less precise than plaque detection. Specifically, the IoU for healthy gingiva was 0.411, whereas for inflamed gingiva was 0.215. The DSC for gingivitis was 70%, precision was 57% and recall 92%.

**Table 2:** Classification performance of the YOLOv8Seg model for plaque and gingival indices (Plaque Index and Gingival Index) on test dataset

Class	Precision	Recall	DSC
Plaque Index	0.983 ±0.010	0.912 ±0.066	0.945 ±0.034
Gingival Index	0.568 ±0.014	0.920 ±0.051	0.702 ±0.025

Precision, recall, and Dice Similarity Coefficient (DSC) values (mean ± standard deviation)

**Fig. 3:**
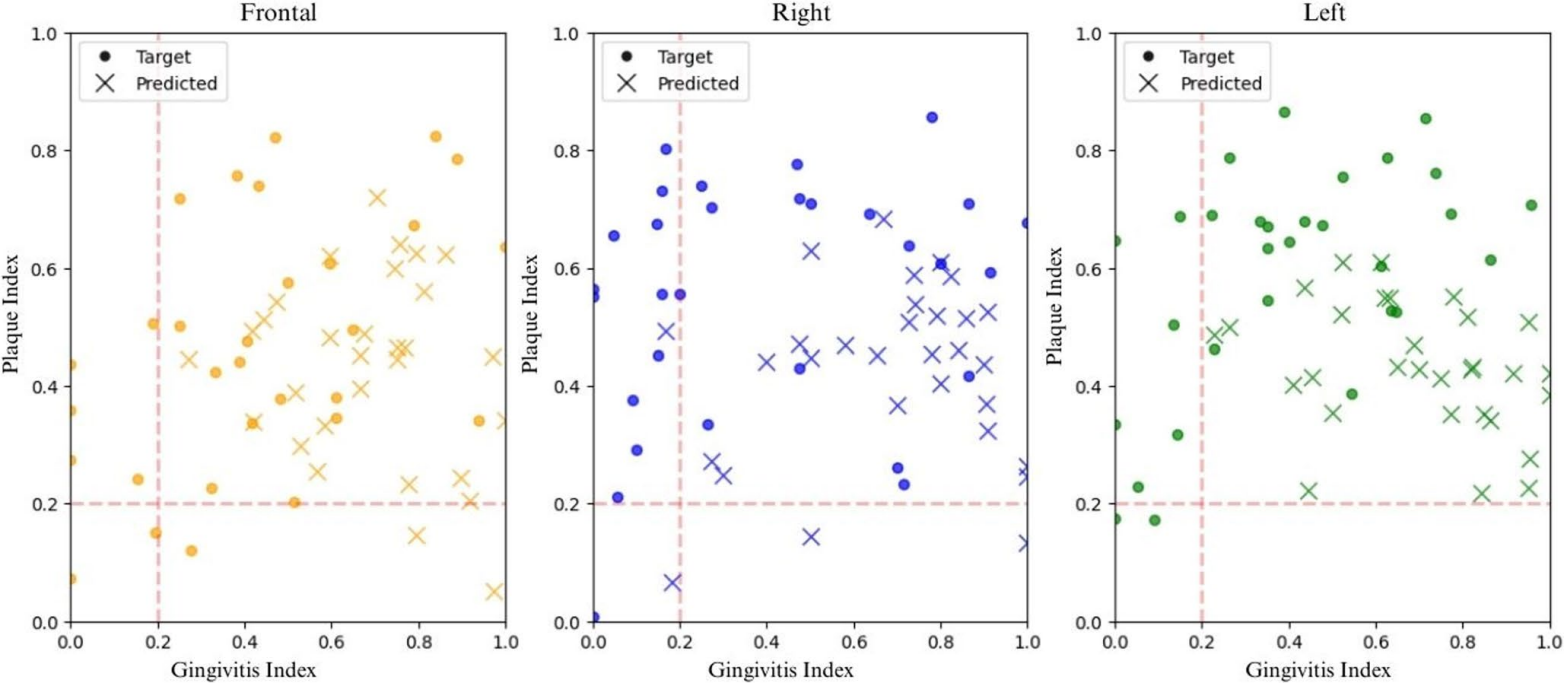
Plaque index versus gingival index in target and predicted masks, frontal and lateral images.Target masks are represented by dots and predicted masks by crosses. Frontal images are shown in yellow, right images in blue, and left images in green

Figure 3 graphically represents the classification performance of the model in relation to the target masks and the predicted masks, categorized by image type (frontal, right, and left). Both overall classification performance was consistent, with frontal images demonstrating better performance compared to lateral images. Additionally, the DSC for plaque index was consistently higher than gingivitis index, reflecting the model’s greater effectiveness in identifying dental biofilm.

**Fig. 4:**
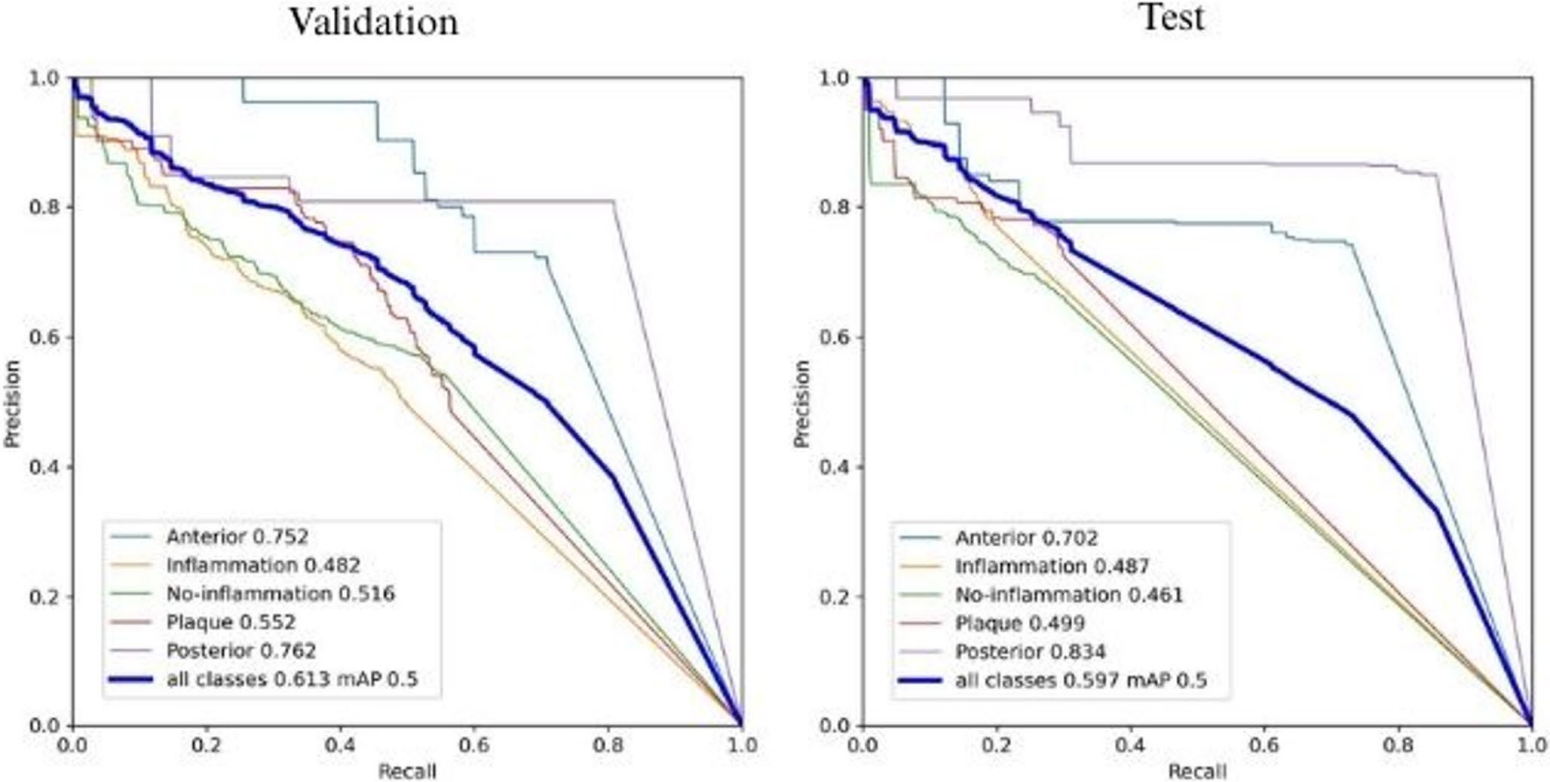
Precision-recall curve in test (T) and validation (V) dataset.Posterior teeth in light blue (VmAP = 0.762, TmAP = 0.834), anterior teeth in purple (VmAP = 0.752, TmAP = 0.702), dental plaque in red (VmAP = 0.552, TmAP = 0.499), healthy gingiva in orange (VmAP = 0.516, TmAP = 0.487), inflamed gingiva in green (VmAP = 0.482, TmAP = 0.487), and overall in cyan (VmAP = 0.613, TmAP = 0.597)

Taking into account the dataset’s imbalance, the model´s performance in correctly detecting each class was evaluated through the precision-versus-recall scatter plot (Fig. 4). The balance between precision and recall was used to select the optimal threshold. Regarding the model’s performance in correctly identifying all classes, the identification of the anterior and posterior tooth areas achieved the best results, followed by the areas with plaque. The model had more difficulty detecting areas with healthy and inflamed gingiva.

###  Qualitative analysis

**Fig. 5:**
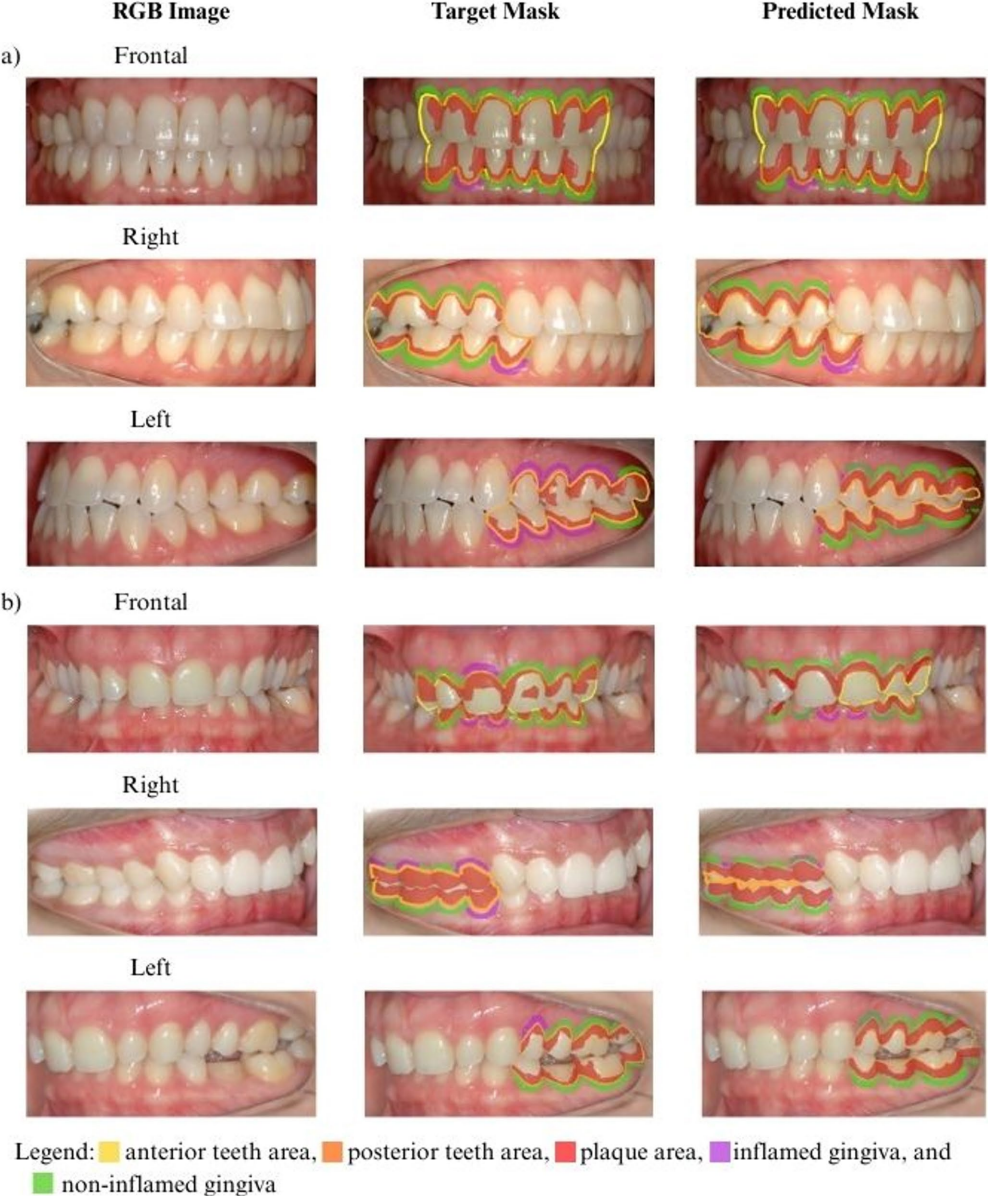
Mosaics of qualitative model performance on the test dataset. The left column is the intraoral image, middle column is the target mask performed by the dentist and the right column is the predicted mask performed by the model. **a** best performance of the model on frontal and lateral view. **b** worst performance of the model on frontal and lateral view. Posterior teeth area in orange, anterior teeth in yellow, dental plaque in red, non-inflamed gingiva in green, inflamed gingiva in purple

The comparison of boundaries marked by the AI (predicted mask) and dentist (target mask) on the RGB image is shown in Fig. 5.

The best results were usually obtained in frontal images with a lower amount of dental biofilm. Usually, the model correctly segmented the biofilm in the cervical third and interproximal regions. The model struggled to correctly detect (1) tooth area in cases of malocclusion (e.g., overbite and overjet), (2) plaque on the mild third of the tooth and (3) areas with signs of inflammation, particularly in lateral images.

##  Discussion

We aimed to develop a patient-centered deep learning model capable of automatically detecting dental plaque and gingival inflammation from intraoral images. The findings demonstrate the feasibility of applying an AI model to automate the assessment of key indicators of oral health. Predictive diagnostics are increasingly recognized as a cornerstone of cost-effective prevention in chronic diseases [27], and our model contributes to this paradigm by providing a non-invasive, image-based tool for early risk recognition in periodontal health. To our knowledge, this is the first study to evaluate a DL method capable of both segmenting and classifying dental biofilm and gingival inflammation in RGB intraoral images.

The analysis demonstrated that an AI algorithm, within a supervised learning environment, could effectively identify high levels of plaque and gingivitis achieving DSC scores of 94% and 70% for plaque and gingivitis, respectively. Evidence indicates that stratifying patients into different risk categories supports tailored interventions and monitoring schedules [5, 28]. The YOLOv8Seg model outputs can be applied to identify individuals requiring intensified preventive care or more frequent professional follow-up, thereby contributing to a personalised maintenance programme.

The model demonstrated moderate performance in segmenting dental biofilm, gingival inflammation, and tooth. Quantitative analysis revealed an average mAP of 60%, with better results for tooth segmentation (posterior mAP of 77% and anterior mAP of 71%), likely due to the comparatively simpler nature of tooth segmentation. In classification tasks, the plaque index performance stood out, achieving a precision of 98%, and recall of 91%. These findings indicate that the model is highly effective in classifying areas with plaque but faces challenges in detecting signs of gingival inflammation with similar accuracy.

Classes with larger pixel-area representation and clearer anatomical boundaries, as anterior and posterior teeth, achieved the highest mAP values, whereas inflamed gingiva, the smallest and most visually heterogeneous class (Suppl. S1), exhibited lower DSC and IoU values. Importantly, despite lower overall segmentation performance in this class, the gingivitis index retained a high recall (92%), meaning the model rarely fails to detect inflammatory sites. Minimising false negatives enhances early predictive detection of inflammation, while occasional false positives reinforce preventive behaviour by prompting users to inspect and improve hygiene in flagged areas.

The similarity observed between the validation and hold-out test precision–recall curves (Fig. 4) further indicates that the dataset offered sufficient variability for the model to generalise to unseen samples. Despite class imbalance and the difficulty of detecting small-area gingival sites, the close proximity of the curves suggests that the dataset composition was adequate. Although external remains necessary for scalability, the internal consistency supports the representativeness of this dataset for early PPPM-oriented applications in oral health.

The quantification and classification of dental biofilm are part of the daily clinical routine of all oral health professionals, whether for disease risk classification, hygiene guidance, or periodontal maintenance protocols. However, these tasks require the presence of an oral health professional. In the last century, research focused on developing indices that accurately describe oral hygiene. In the early 2000 s, with the popularisation of digital photography, research shifted towards automating dental biofilm evaluation, aiming to bring reproducibility and objectivity to what remains a subjective process in practice. In recent years, the focus has shifted towards developing methodologies that leverage digital images integrated with new technologies such as artificial intelligence. The proposed AI method demonstrated performance comparable to other models [14, 29] and supports the development of targeted prevention strategies, since site-specific identification of biofilm allows more precise guidance on oral hygiene practices, providing to patients an objective visual feedback on areas requiring improved cleaning, the model reinforces behavioural modification and encourages adherence to preventive routines.

Additionally, the model consistently captured the expected biological relationship between plaque accumulation and inflammatory burden. Cases presenting high plaque index almost invariably exhibited higher predicted gingivitis index values, even in lateral views where gingival sites are smaller and more challenging to segment (Fig. 3). This reflects not only the model’s ability to detect visible plaque but also its capacity to represent clinically meaningful patterns. Such behaviour strengthens its applicability for personalised monitoring, risk stratification, and timely preventive action.

The analysis was conducted comprehensively, evaluating all present teeth, whether deciduous or permanent, rather than focusing on individual teeth. Although dental plaque can be detected in isolated tooth images using DL models, some approaches rely on disclosing agents or high-resolution images [13, 30] to enhance detection, which may limit the applicability in self-monitoring contexts. The ability to assess multiple teeth and gingival regions simultaneously using routine intraoral photographs represents an important advancement in predictive modelling, as it enables early identification of sites with higher inflammatory burden before clinical symptoms become pronounced. By providing automated, objective, and reproducible quantification of plaque and gingival inflammation without the need for adjunctive agents, the model supports preventive strategies aimed at reducing biofilm accumulation and interrupting disease progression at an early stage. Moreover, the segmentation outputs can guide personalised recommendations for oral hygiene reinforcement, with potential integration into participatory digital health monitoring frameworks that empower patients to track their own risk profile over time. Together, these elements position the model as a practical tool for operationalising predictive and preventive dental care within a PPPM framework.

Quantitative and qualitative analysis showed that the presented model was able to detect the disease areas, had limited success in capturing complete segmentation masks, which explains why disease segmentation metrics were not higher. Regarding dental biofilm, as with Andrade et al.‘s [14] study, which reported a DSC of 61% with a U-Net network, we encountered challenges in accurately delineating the biofilm boundaries, as also observed in the qualitative analysis (Fig. 4). A YOLOv8Seg detection model using intraoral images achieved a DSC score of 77% but applied stricter exclusion criteria, including plaque disclosing agents and targeting specific teeth [31, 32], factors that may limit its applicability in real-world oral hygiene self-monitoring contexts.

Annotating the 504 images consumed significant research effort, totalling 480 h of work. Besides, AI models typically require larger datasets [33]; a five-fold cross-validation approach was integrated to enhance prediction performance. Although only one examiner annotated the images, potentially introducing rater bias, a single examiner with expertise in periodontics, computer vision, and AI, the use of a single, expert examiner helped enhance the consistency and robustness of the annotations [17].

Most studies in the medical field rely on metrics such as sensitivity and specificity; however, we did not consider these measures. While sensitivity and specificity are considered gold standard metrics in diagnostic studies, AI models require more robust metrics that reflect the agreement between predicted and target classes, and account for potential imbalanced number of real positive and negative instances [24]. Metrics that use True Negative (TN) confusion matrix values were excluded due to the large number of TN predictions and background pixels in the images, which can skew metrics that rely on TN counts. The substantial or undefined number of TN predictions in segmentation and object detection tasks causes TN focused metrics to appear artificially high, despite the model’s true performance. Furthermore, as this model is designed to be used by non-expert users to identify areas of poor hygiene, it is preferable for the model to prioritise FP rather than FN. This contrasts with other medical domains, where a higher number of FN predictions is preferred to prevent overdiagnosis and overtreatment [34].

This study does not aim to replace clinical diagnosis, but rather to empower patients by facilitating early self-detection of plaque and gingival inflammation. Holistic approaches in PPPM, emphasised that AI-based image analysis should not replace comprehensive periodontal assessment. Instead, such tools should be integrated with microbial, behavioural, and socioeconomic data to generate a more complete patient profile, reinforcing their role in predictive diagnostics, targeted prevention, and personalised treatment planning [35]. Our findings align with PPPM principles recently advocated for dentistry, in which patient stratification and tailored maintenance are central to proactive care. Dental care tailored to the person, integrating risk profiles and cost-effective prevention at population level, represents a cornerstone of this paradigm [36]. By quantifying site-specific plaque and gingival status and operationalising threshold-based alerts, our approach provides actionable strata that map directly onto targeted prevention and timely referral, thereby supporting the paradigm shift towards PPPM-oriented periodontal care.

Periodontal diseases are increasingly recognised as part of a broader chronic inflammatory network with systemic implications, including metabolic, cardiovascular, and immune-mediated pathways. Both periodontal disease and dental caries share fundamental behavioural and lifestyle determinants such as oral hygiene behaviour and socioeconomic factors, supporting the interpretation of plaque control as a modifiable risk factor of systemic relevance [37]. Within this framework, AI-derived digital biomarkers extend beyond local oral disease monitoring by enabling early identification of individuals with elevated inflammatory burden. Integrating image-based outputs with microbial, metabolic, behavioural and socioeconomic data [38–40] supports multi-level diagnostics and deeper patient phenotyping. This systems-oriented perspective reinforces the role of AI tools in anticipatory care, where early stratification of risk can guide targeted preventive strategies and personalised intervention pathways before clinical deterioration occurs.

Although the model demonstrated capability in detecting poor oral hygiene and inadequate gingival indices, this study had limitations, and further research in this field is needed. Future studies should focus on associating intraoral images with oral diseases without relying on indices developed for clinical practice. Moreover, the use of standardised images in controlled environments (e.g., saliva and lighting conditions) could hinder real-world implementation, despite our focus on developing a lightweight model suitable for smartphone applications. Longitudinal clinical studies assessing the use of smartphone apps for oral hygiene and gingivitis self-monitoring are crucial for improving the performance of these models. It is essential to identify the areas with dental biofilm and gingival inflammation, and more importantly, demonstrate the physical proximity between biofilm and gingivitis signs, reinforcing their potential role in reducing the incidence and progression of plaque-induced gingivitis and periodontitis.

From a broader PPPM perspective, AI-driven intraoral analysis also contributes to patient phenotyping and multi-level risk stratification by capturing early markers of chronic inflammation and linking them to behavioural and structural determinants of oral health. The integration of imaging biomarkers with multi-parametric data, such as microbial profiles, lifestyle indicators, and socioeconomic risk factors, enables a holistic characterisation of patient trajectories rather than isolated disease episodes [5, 36, 38–40]. Such a systems-oriented approach recognises periodontal inflammation as part of a wider chronic inflammatory network, reinforcing the need for predictive modelling that identifies high-risk phenotypes before clinical deterioration. The proposed model provides a foundation for this integrative strategy by generating standardised digital biomarkers suitable for longitudinal digital health monitoring, allowing early alerts, dynamic adjustment of preventive plans, and personalised thresholds for intervention.

##  Conclusions and expert recommendations

This study demonstrates the feasibility of using a lightweight, patient-centred deep learning model to detect dental plaque and gingival inflammation from routine intraoral photographs, offering a practical pathway toward predictive and preventive periodontal care. Rather than replacing clinical examination, the model strengthens early risk recognition by providing objective, image-based markers of biofilm accumulation and inflammatory burden. Such functionality aligns with PPPM principles by enabling stratification of individuals according to their site-specific risk, supporting timely behavioural reinforcement, and facilitating personalised maintenance strategies.

### Expert recommendations

1. Integration into patient-facing digital tools
  Embedding AI-based plaque and gingivitis assessment into mobile health applications may strengthen participatory engagement and enable individuals to track longitudinal changes in their oral health.
2. Multimodal clinical use
  For professional environments, AI outputs should be combined with behavioural, microbiological, and socioeconomic information to support more accurate risk stratification and comprehensive patient profiling.
3. Dataset expansion and longitudinal validation
  Future research should include multi-centre cohorts and follow-up studies to refine predictive accuracy and test generalisability across diverse populations.
4. Operationalising targeted prevention
  Incorporating site-level alerts and threshold-driven risk categories can help clinicians tailor recall intervals, oral hygiene instruction, and preventive interventions.
5. Public health implementation
  Deploying lightweight AI tools in community-based programmes may enhance early identification of plaque-induced conditions and reduce the long-term burden of preventable periodontal disease.

#### Innovation towards the predictive approach, targeted prevention, and personalisation

The proposed model supports the three pillars of PPPM by enabling early prediction of periodontal risk, guiding targeted preventive action, and allowing personalised self-monitoring through scalable, real-world digital tools. These functionalities align with recent PPPM recommendations in dentistry, which emphasise early risk stratification and personalised maintenance pathways [5, 36, 38, 40]. By transforming image-based findings into actionable risk strata, the system accelerates the shift from reactive treatment to proactive, precision-driven oral healthcare.

## Supplementary Information

Below is the link to the electronic supplementary material.

Supplementary Material 1 (DOCX 6.43 MB)

Supplementary Material 2 (DOCX 16.4 KB)

Supplementary Material 3 (PDF 26.0 KB)

## Data Availability

The datasets generated and/or analyzed during the current study are available from the corresponding author on reasonable request.

## References

[1] Amarasena N, Chrisopoulos S, Jamieson LM, Luzzi L. Oral Health of Australian Adults: distribution and time trends of dental caries, periodontal disease and tooth loss. Int J Environ Res Public Health. 2021;18(21):11539. 10.3390/ijerph182111539. 34770052 10.3390/ijerph182111539 PMC8583389

[2] Marsh PD, Zaura E. Dental biofilm: ecological interactions in health and disease. J Clin Periodontol. 2017;44(Suppl 18):S12–22. 10.1111/jcpe.12679. 28266111 10.1111/jcpe.12679

[3] Kimura ACRS, de Arruda JAA, Drumond VZ, Martins-Júnior PA, Mesquita RA, Abreu LG. Dental caries and periodontal outcomes in Mouth-Breathing children and adolescents: A systematic review. Int J Paediatr Dent. 2025;30. 10.1111/ipd.70022. 10.1111/ipd.70022 40739849

[4] Cafiero C, Matarasso S. Predictive, preventive, personalised and participatory periodontology: “the 5Ps age” has already started. EPMA J. 2013;4(1):16. 10.1186/1878-5085-4-16. 23763842 10.1186/1878-5085-4-16 PMC3703280

[5] Tachalov VV, Orekhova LY, Kudryavtseva TV, Loboda ES, Pachkoriia MG, Berezkina IV, Golubnitschaja O. Making a complex dental care tailored to the person: population health in focus of predictive, preventive and personalised (3P) medical approach. EPMA J. 2021;12(2):129–40. 10.1007/s13167-021-00240-7. 33897916 10.1007/s13167-021-00240-7 PMC8053896

[6] Slots J. Periodontitis: facts, fallacies and the future. Periodontol 2000. 2017;75(1):7–23. 10.1111/prd.12221. 28758294 10.1111/prd.12221

[7] Van der Weijden GAF, van Loveren C. Mechanical plaque removal in step-1 of care. Periodontol 2000;2023. 10.1111/prd.12541 10.1111/prd.12541 38148481

[8] Tonetti MS, Eickholz P, Loos BG, Papapanou P, van der Velden U, Armitage G, et al. Principles in prevention of periodontal diseases: consensus report of group 1 of the 11th European Workshop on Periodontology on effective prevention of periodontal and peri-implant diseases. J Clin Periodontol. 2015;42(Suppl 16):S5-11. 10.1111/jcpe.12368. 25639948 10.1111/jcpe.12368

[9] Azevedo CL, Henriques PS, Pannuti CM, Michel-Crosato E. Selfie dental plaque index: a new tool for dental plaque assessment. J Clin Exp Dent. 2022;14(11):e926-31. 10.4317/jced.59908. 36458034 10.4317/jced.59908 PMC9701342

[10] Kasai M, Iijima Y, Takemura H, Mizoguchi H, Ohshima T, Satomi N. Dental plaque assessment lifelogging system using commercial camera for oral healthcare. 38th Annual International Conference of the IEEE Engineering in Medicine and Biology Society (EMBC). Orlando, FL, USA. 2016, pp. 2566–2569. 10.1109/EMBC.2016.7591254 10.1109/EMBC.2016.7591254 28268846

[11] Khanagar SB, Al-Ehaideb A, Maganur PC, Vishwanathaiah S, Patil S, Baeshen HA, et al. Developments, application, and performance of artificial intelligence in dentistry - a systematic review. J Dent Sci. 2021;16(1):508–22. 10.1016/j.jds.2020.06.019. 33384840 10.1016/j.jds.2020.06.019 PMC7770297

[12] Yauney G, Angelino K, Edlund D, Shah P. Convolutional neural network for combined classification of fluorescent biomarkers and expert annotations using white light images. 17th International Conference on Bioinformatics and Bioengineering; 2017; Washington, DC, USA. [cited 2025 Jan 28]. Available from: https://ieeexplore.ieee.org/document/8251307

[13] You W, Hao A, Li S, Wang Y, Xia B. Deep learning-based dental plaque detection on primary teeth: a comparison with clinical assessments. BMC Oral Health. 2020;20(1):141. 10.1186/s12903-020-01114-6. 32404094 10.1186/s12903-020-01114-6 PMC7222297

[14] Andrade KM, Silva BPM, de Oliveira LR, Cury PR. Automatic dental biofilm detection based on deep learning. J Clin Periodontol. 2023;50(5):571–81. 10.1111/jcpe.13774. 36635042 10.1111/jcpe.13774

[15] Chen Y, Chen X. Gingivitis identification via GLCM and artificial neural network. In: Su R, Liu H, editors. Medical Imaging and Computer-Aided Diagnosis: Proceedings of the 2020 International Conference on Medical Imaging and Computer-Aided Diagnosis (MICAD 2020). Lecture Notes in Electrical Engineering, vol. 633. Singapore: Springer; 2020. p. 95–106. 10.1007/978-981-15-5199-4

[16] Chau RCW, Li GH, Tew IM, Thu KM, McGrath C, Lo WL, et al. Accuracy of artificial intelligence-based photographic detection of gingivitis. Int Dent J. 2023;73(5):724–30. 10.1016/j.identj.2023.03.007. 37117096 10.1016/j.identj.2023.03.007 PMC10509417

[17] Li W, Liang Y, Zhang X, Liu C, He L, Miao L, Sun W. A deep learning approach to automatic gingivitis screening based on classification and localization in RGB photos. Sci Rep. 2021;11(1):16831. 10.1038/s41598-021-96091-3. 34413332 10.1038/s41598-021-96091-3 PMC8376991

[18] Alalharith DM, Alharthi HM, Alghamdi WM, Alsenbel YM, Aslam N, Khan IU, et al. A deep learning-based approach for the detection of early signs of gingivitis in orthodontic patients using faster region-based convolutional neural networks. Int J Environ Res Public Health. 2020;17(22):8447. 10.3390/ijerph17228447. 33203065 10.3390/ijerph17228447 PMC7697132

[19] Reis D, Hong J, Kupec J, Daoudi A. Real-time flying object detection with YOLOv8.[Internet]. 2024 May 22 [revised 2024; cited 2025 Jan 28]. Available from: https://arxiv.org/abs/2305.09972

[20] Schwendicke F, Singh T, Lee JH, Gaudin R, Chaurasia A, Wiegand T, et al. IADR e-oral health network and the ITU WHO focus group AI for health. Artificial intelligence in dental research: checklist for authors, reviewers, readers. J Dent. 2021;107:103610. 10.1016/j.jdent.2021.103610.

[21] Dutta A, Zisserman A, ACM International Conference on Multimedia (MM ’19). The VIA Annotation Software for Images, Audio and Video. 27th. 2019 October 21–25, Nice, France; ACM, New York, NY, USA. 10.1145/3343031.3350535

[22] Terven J, Cordova-Esparza D. A comprehensive review of YOLO architectures in computer vision: From YOLOv1 to YOLOv8 and YOLO-NAS.[Internet]. 2024 Feb 4 [cited 2025 Jan 28]. Available from: arXiv:2304.00501.

[23] Li G, Huang Y, Chen Z, Chesser GD Jr, Purswell JL, Linhoss J, et al. Practices and applications of convolutional neural network-based computer vision systems in animal farming: a review. Sensors. 2021;21(4):1492. 10.3390/s21041492. 33670030 10.3390/s21041492 PMC7926480

[24] Rainio O, Teuho J, Klén R. Evaluation metrics and statistical tests for machine learning. Sci Rep. 2024;14(1):6086. 10.1038/s41598-024-56706-x. 38480847 10.1038/s41598-024-56706-x PMC10937649

[25] Reiniger APP, Maier J, Wikesjö UME, Moreira CHC, Kantorski KZ. Correlation between dental plaque accumulation and gingival health in periodontal maintenance patients using short or extended personal oral hygiene intervals. J Clin Periodontol. 2021;48(6):834–42. 10.1111/jcpe.13448. 33751652 10.1111/jcpe.13448

[26] Azevedo CL, Silva LRV, Alencar CO, Braga MM, Biazevic MGH, Michel-Crosato E. Is there a safe dental plaque index to prevent periodontal diseases related to plaque? A systematic review and meta-analysis. Res Soc Dev. 2022;11(7):e27511730100. 10.33448/rsd-v11i7.30100.

[27] Golubnitschaja O, Liskova A, Koklesova L, Samec M, Biringer K, Büsselberg D, et al. Caution, “normal” BMI: health risks associated with potentially masked individual underweight-EPMA Position Paper 2021. EPMA J. 2021;12(3):243–64. 10.1007/s13167-021-00251-4. 34422142 10.1007/s13167-021-00251-4 PMC8368050

[28] Ma X, Wang Y, Wu H, Li F, Feng X, Xie Y, Xie D, Wang W, Lo ECM, Lu H. Periodontal health related-inflammatory and metabolic profiles of patients with end-stage renal disease: potential strategy for predictive, preventive, and personalized medicine. EPMA J. 2021;12(2):117–28. 10.1007/s13167-021-00239-0. 33903806 10.1007/s13167-021-00239-0 PMC8060784

[29] Revilla-León M, Gómez-Polo M, Barmak AB, Inam W, Kan JYK, Kois JC, et al. Artificial intelligence models for diagnosing gingivitis and periodontal disease: a systematic review. J Prosthet Dent. 2023;130(6):816–24. 10.1016/j.prosdent.2022.01.026. 35300850 10.1016/j.prosdent.2022.01.026

[30] Nantakeeratipat T, Apisaksirikul N, Boonrojsaree B, Boonkijkullatat S, Simaphichet A. Automated machine learning for image-based detection of dental plaque on permanent teeth. Front Dent Med. 2024;5:1507705. 10.3389/fdmed.2024.1507705. 39917656 10.3389/fdmed.2024.1507705 PMC11797812

[31] Chen X, Shen Y, Jeong JS, Perinpanayagam H, Kum KY, Gu Y. DeepPlaq: dental plaque indexing based on deep neural networks. Clin Oral Investig. 2024;28(10):534. 10.1007/s00784-024-05921-x. 39302479 10.1007/s00784-024-05921-x

[32] Yüksel B, Özveren N, Yeşil Ç. Evaluation of dental plaque area with artificial intelligence model. Niger J Clin Pract. 2024;27(6):759–65. 10.4103/njcp.njcp_862_23. 38943301 10.4103/njcp.njcp_862_23

[33] Wataya T, Nakanishi K, Suzuki Y, Kido S, Tomiyama N. Introduction to deep learning: minimum essence required to launch a research. Jpn J Radiol. 2020;38(10):907–21. 10.1007/s11604-020-00998-2. 32556733 10.1007/s11604-020-00998-2

[34] Šimundić AM. Measures of diagnostic accuracy: basic definitions. EJIFCC. 2009;19(4):203–11. PMC4975285 27683318

[35] Kunin A, Polivka J Jr, Moiseeva N, Golubnitschaja O. Dry mouth" and “Flammer” syndromes-neglected risks in adolescents and new concepts by predictive, preventive and personalised approach. EPMA J. 2018;9(3):307–17. 10.1007/s13167-018-0145-7. 30174766 10.1007/s13167-018-0145-7 PMC6107455

[36] Pei J, Li F, Xie Y, Liu J, Yu T, Feng X. Microbial and metabolomic analysis of gingival crevicular fluid in general chronic periodontitis patients: lessons for a predictive, preventive, and personalized medical approach. EPMA J. 2020;11(2):197–215. 10.1007/s13167-020-00202-5. 32547651 10.1007/s13167-020-00202-5 PMC7272536

[37] Chapple IL, Bouchard P, Cagetti MG, Campus G, Carra MC, Cocco F, et al. Interaction of lifestyle, behaviour or systemic diseases with dental caries and periodontal diseases: consensus report of group 2 of the joint EFP/ORCA workshop on the boundaries between caries and periodontal diseases. J Clin Periodontol. 2017;44(18):S39–51. 10.1111/jcpe.12685. 28266114 10.1111/jcpe.12685

[38] Smokovski I, Steinle N, Behnke A, Bhaskar SMM, Grech G, Richter K, Niklewski G, Birkenbihl C, Parini P, Andrews RJ, Bauchner H, Golubnitschaja O. Digital biomarkers: 3PM approach revolutionizing chronic disease management - EPMA 2024 position. EPMA J. 2024;15(2):149–62. 10.1007/s13167-024-00364-6. 38841615 10.1007/s13167-024-00364-6 PMC11147994

[39] Oh E, Jun JH, Choi JY, Yoo TK. Systemic inflammation at the oral-ocular interface: a 3P medicine perspective on the relationship between periodontitis and eye diseases. EPMA J. 2025;16(3):571–87. 10.1007/s13167-025-00415-6. 40948984 10.1007/s13167-025-00415-6 PMC12423021

[40] Golubnitschaja O. How to use an extensive flammer syndrome phenotyping for a holistic protection against health-to-disease transition - facts and practical recommendations. EPMA J. 2025;16(3):535–9. 10.1007/s13167-025-00423-6. 40948987 10.1007/s13167-025-00423-6 PMC12422999

