# Supplementary material for: AI-enabled predictive, preventive and personalised oral health management: a lightweight patient-centred model for automated assessment of dental plaque and gingival inflammation
Source: EPMA J. 2026 Feb 24;17(1):43–55. doi: 10.1007/s13167-025-00432-5 (PMC12976221; doi:10.1007/s13167-025-00432-5)
Supplement: Supplementary file 1 — (DOCX 6.43 MB) [file 13167_2025_432_MOESM1_ESM.docx]

**Project Workflow**

**Project name:** Dental plaque and gingival inflammation prediction

**Description:** Classify periodontal status and oral hygiene status

**Introduction:** Periodontal diseases can cause tooth loss and are related to systemic diseases like diabetes and atherosclerotic vascular diseases. Primary prevention should be focused on health education and plaque control. The detection of dental plaque and gingival inflammation could be a valuable tool for population level interventions. We need an experienced dental professional to teach for the A.I. how a healthy mouth should be.

**Manual Annotations:**

1. RGB Image
   1. Identification
      1. Anterior teeth area: polygon region shape
      2. Posterior teeth area: polygon region shape
      3. Dental plaque area: polygon region shape
      4. Stain area (tobacco/others): polygon region shape
      5. Non-inflammation gingival site: polygon region shape
      6. Inflammation gingival site: polygon region shape
   2. View
      1. Visible (frontal images: anterior teeth, left/right images: posterior teeth)
      2. Not visible (frontal images: posterior teeth, left/right images: anterior teeth)

**Annotation Guide**

Software: <https://www.robots.ox.ac.uk/~vgg/software/via/via_demo.html>

You can save your progress on a .json file (for continue later).

Workflow 1 (RGB image)

1. Load the project. Image path and attributes. Ex: Workflow_1(001-020).json


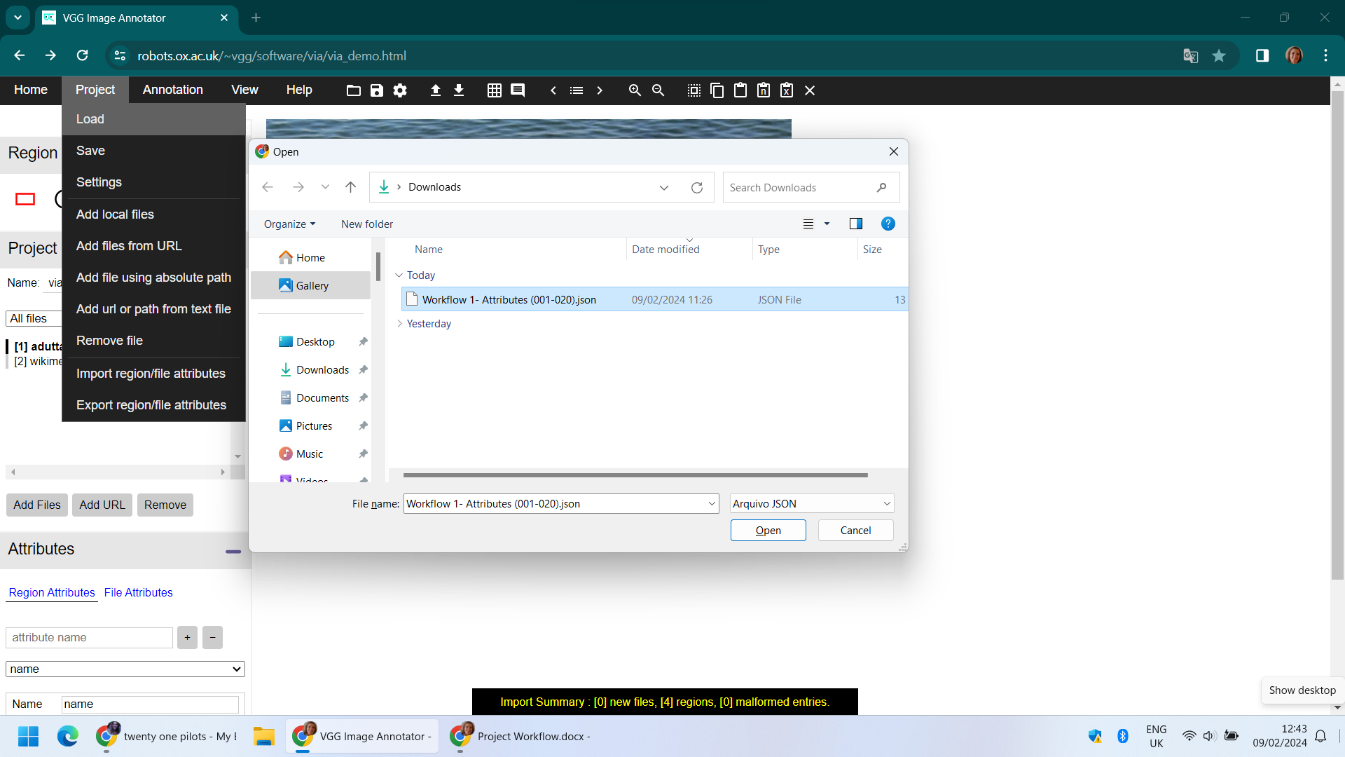


1. After load the .json file:


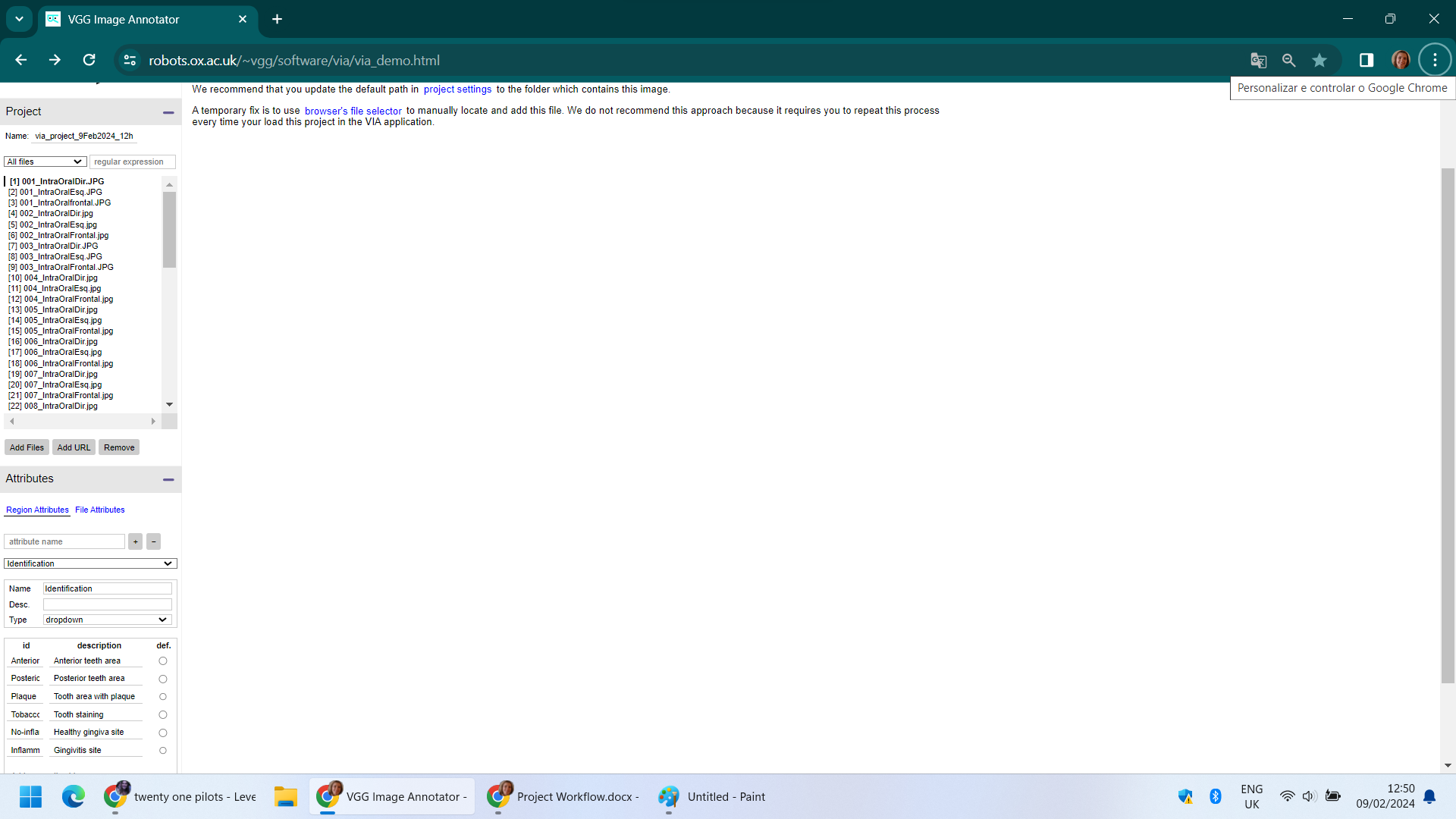



Images to be annotated



 Project attributes

1. Load the images. Ex: folder “Workflow 1_001-020"


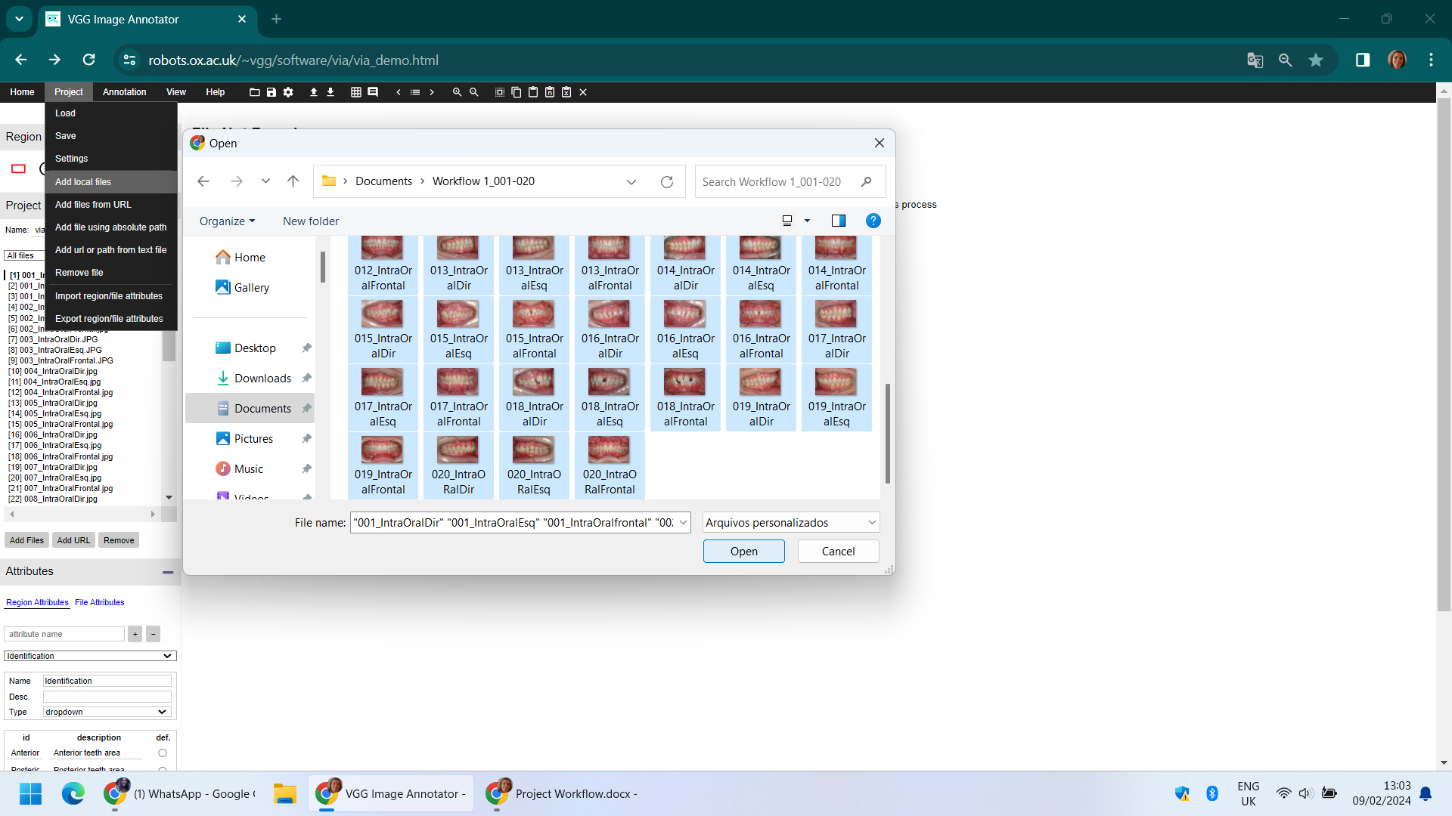


1. Name your project (“Project Name”) with your examiner's initials and folder’s name, separate with underscore. Ex: CLA_Workflow 1_001-020


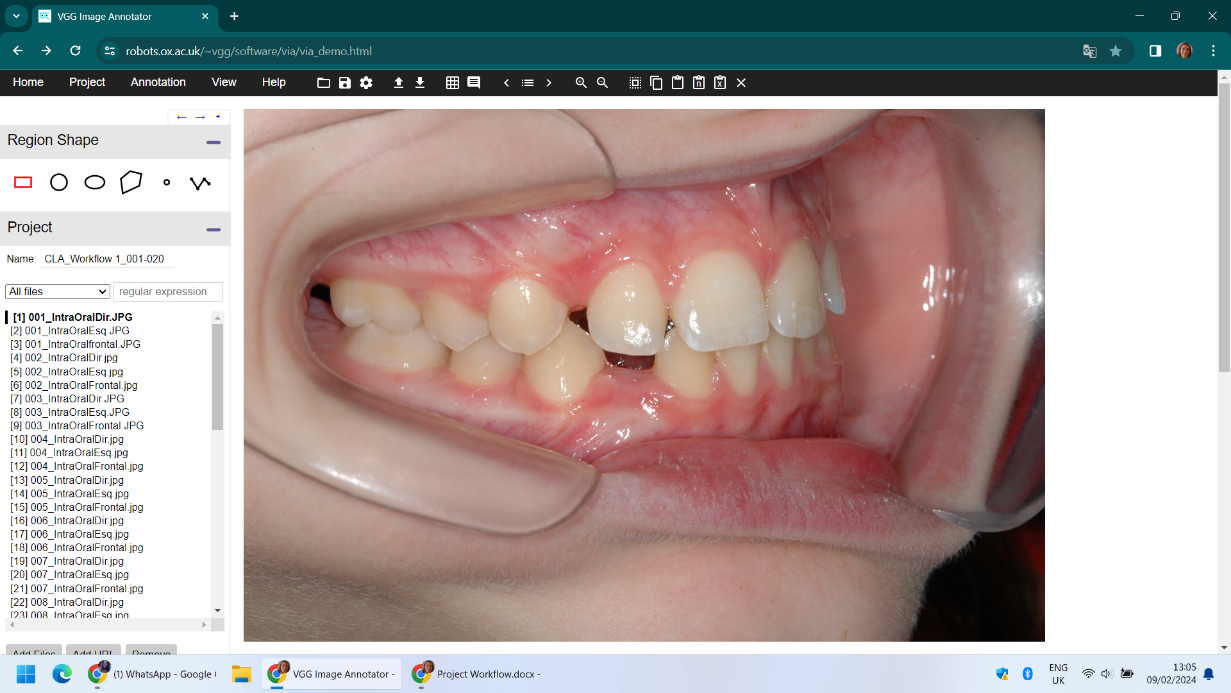



1. Plaque area detection.
   1. Select the polygon region shape.
   2. You can use Zoom In/Out.
   3. Label the selected area clicking above it (Identification=” tooth area with plaque”, View= “classify area”/”non-classifiable area”).
      1. Frontal image: anterior teeth are visible and must be annotated as “classify area”, posterior teeth are not completely visible and must be annotated as ”non-classifiable area”.
      2. Lateral images (left and right side): anterior teeth are not completely visible and must be annotated as ”non-classifiable area”, posterior teeth are visible and must be annotated as “classify area”.
   4. Select as many areas as needed.


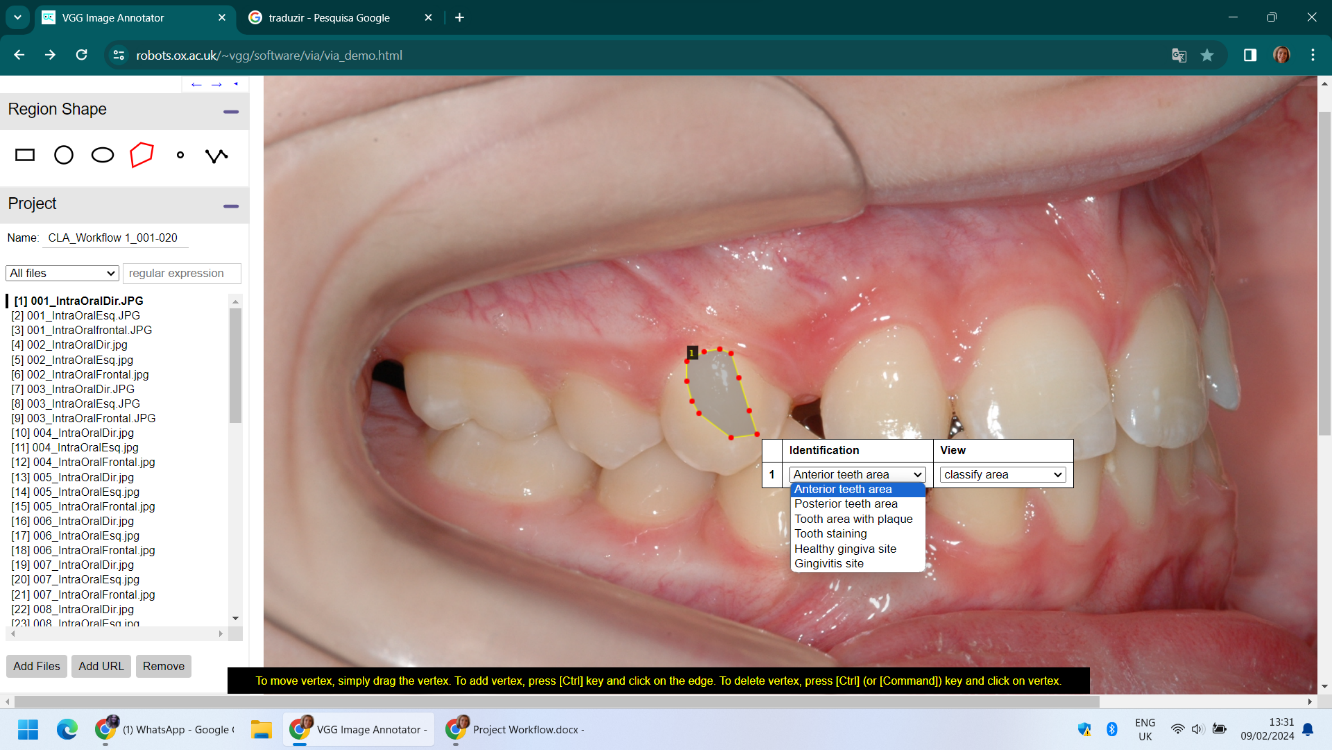


1. Stain area detection .
   1. Select the polygon region shape.
   2. You can use Zoom In/Out.
   3. Label the selected area clicking above it (Identification=” tooth staining”, View= “classify area” /”non-classifiable area”).
      1. Frontal image: anterior teeth are visible and must be annotated as “classify area”, posterior teeth are not completely visible and must be annotated as ”non-classifiable area”.
      2. Lateral images (left and right side): anterior teeth are not completely visible and must be annotated as ”non-classifiable area”, posterior teeth are visible and must be annotated as “classify area”.
   4. Select as many areas as needed.
2. Teeth area detection
   1. Select the polygon region shape.
   2. You can use Zoom In/Out.
   3. Select the area of ​​the anterior teeth and the area of ​​the posterior teeth, separately.
   4. Label the selected area clicking above it. Example: Identification=” Anterior teeth area”, View= “classify area”, if it is a frontal image.


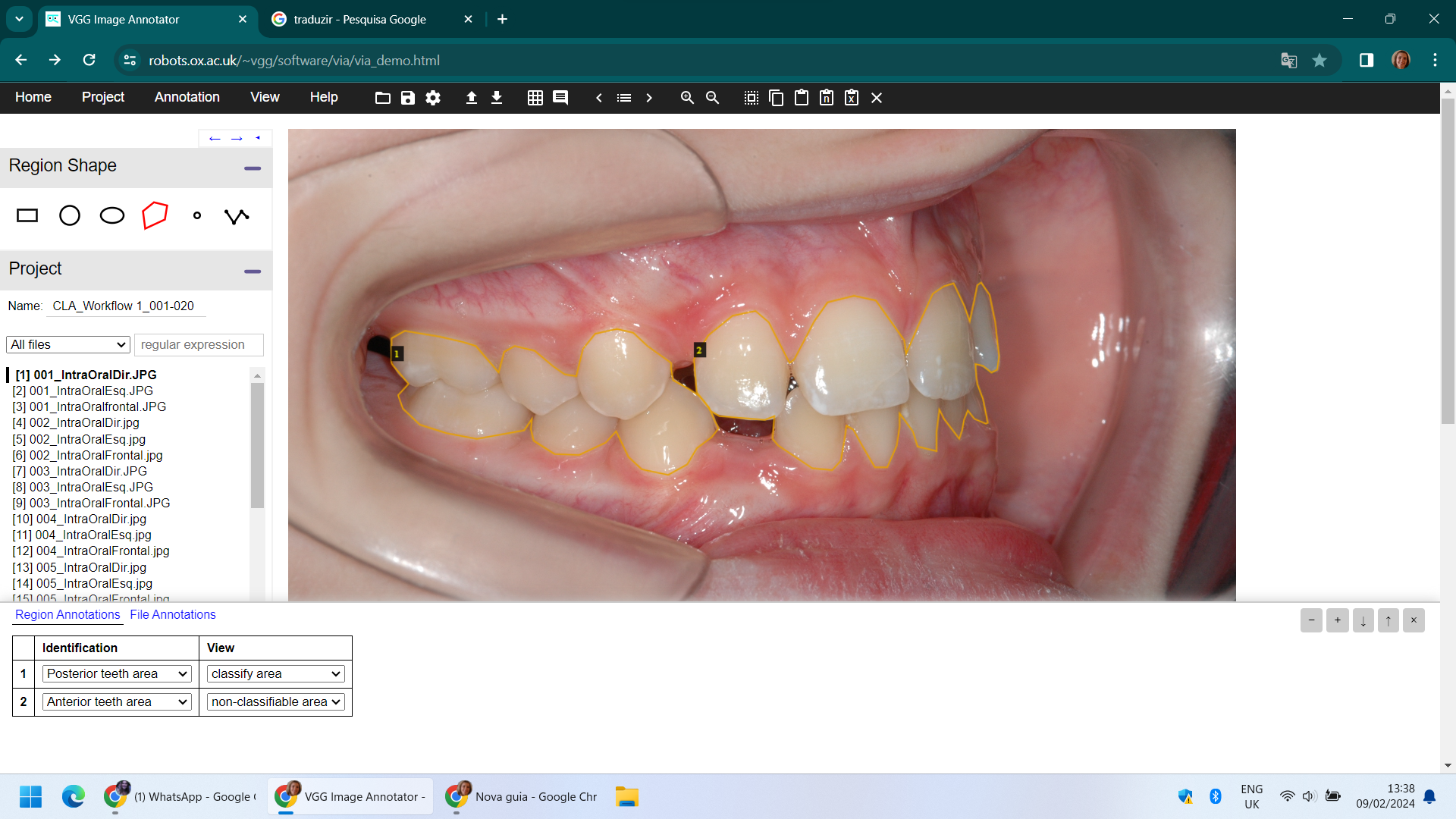


1. Gingival status
   1. Three gingival sites per tooth (mesial, mid, distal).
   2. Label the selected area clicking above it. Label the area with (gingivitis site) or without (healthy gingival site) inflammation signs through “Identification” dropdown. The view should be label with “non classifiable” or “classify area”, depending on image type (frontal or right/left side).
      1. Frontal image: anterior teeth are visible and must be annotated as “classify area”, posterior teeth are not completely visible and must be annotated as ”non-classifiable area”.
      2. Lateral images (left and right side): anterior teeth are not completely visible and must be annotated as ”non-classifiable area”, posterior teeth are visible and must be annotated as “classify area”.


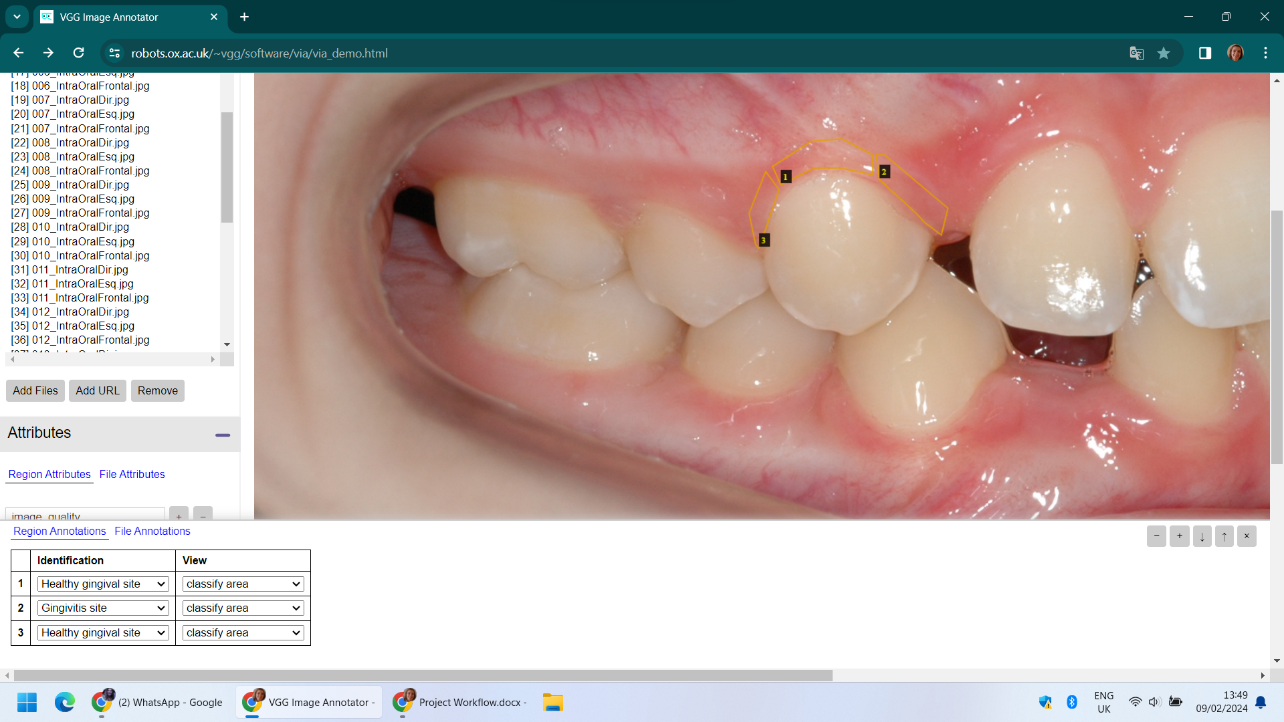


1. When the annotation process was concluded, export and send the annotations in .json and .csv
