# Supplementary material for: AI-enabled predictive, preventive and personalised oral health management: a lightweight patient-centred model for automated assessment of dental plaque and gingival inflammation
Source: EPMA J. 2026 Feb 24;17(1):43–55. doi: 10.1007/s13167-025-00432-5 (PMC12976221; doi:10.1007/s13167-025-00432-5)
Supplement: Supplementary file 2 — (DOCX 16.4 KB) [file 13167_2025_432_MOESM2_ESM.docx]

**Per-class counts and balance ratios**

| Supplementary Table. Per-class instance and per image pixel counts for the 504-image dataset. | | | | | | |
| --- | --- | --- | --- | --- | --- | --- |
| **Class** | **Background** | **Anterior** | **Inflammation** | **No-Inflammation** | **Plaque** | **Posterior** |
| **Class Number** | - | 0 | 1 | 2 | 3 | 4 |
| **Instance Count** | - | 2,202 | 10,015 | 18,726 | 4,342 | 1,706 |
| **Pixel Count** | 3,311,364 | 249,741 | 88,923 | 112,208 | 429,050 | 164,914 |
