# Supplementary material for: AI-enabled predictive, preventive and personalised oral health management: a lightweight patient-centred model for automated assessment of dental plaque and gingival inflammation
Source: EPMA J. 2026 Feb 24;17(1):43–55. doi: 10.1007/s13167-025-00432-5 (PMC12976221; doi:10.1007/s13167-025-00432-5)
Supplement: Supplementary file 3 — (PDF 26.0 KB) [file 13167_2025_432_MOESM3_ESM.pdf]

PARECER CONSUBSTANCIADO DO CEP

DADOS DO PROJETO DE PESQUISA

Título da Pesquisa: UTILIZAÇÃO DE REDES NEURAIS (MACHINE LEARNING) NA PREDIÇÃO DE DOENÇAS ORAIS E IDENTIFICAÇÃO DE ESTRUTURAS ANATÔMICAS DE INTERESSE ODONTOLÓGICO

Pesquisador: Camila Lindoni Azevedo

Área Temática:

Versão: 2

CAAE: 62922922.9.0000.0075

Instituição Proponente: Faculdade de Odontologia da Universidade de São Paulo

Patrocinador Principal: Financiamento Próprio

DADOS DA NOTIFICAÇÃO

Tipo de Notificação: Envio de Relatório Parcial

Detalhe:

Justificativa: Atraso no cronograma e atualizações no status da pesquisa.

Data do Envio: 13/05/2024

Situação da Notificação: Parecer Consubstanciado Emitido

DADOS DO PARECER

Número do Parecer: 6.865.095

Apresentação da Notificação:

Trata-se de Relatório Parcial da Pesquisa.

Objetivo da Notificação:

Trata-se de Relatório Parcial da Pesquisa.

Avaliação dos Riscos e Benefícios:

Trata-se de Relatório Parcial da Pesquisa.

Comentários e Considerações sobre a Notificação:

Trata-se de Relatório Parcial da Pesquisa onde a pesquisadora responsável coloca as seguintes justificativas:

Resultados alcançados:
"Coleta de Dados: 800 documentações totalmente anonimizadas
Rotulagem: 500 imagens RGB identificadas em relação a biofilme dental, condição gengival e dentes.
Rede neural: Teste com uma amostra de 200 imagens RGB com DICE médio (acurácia) de 0.9 para identificação dos dentes e de biofilme dental."

"Houve necessidade de reorganizar o cronograma e rever parcerias para o desenvolvimento do algoritmo. Através de um edital de Doutorado Sanduiche da Capes, estamos desenvolvendo uma nova rede neural, objetivando a predição de biofilme dental, inflamação gengival (com as imagens intraorais) e perda óssea radiográfica (com a radiografia panorâmica). Para aumentar validade da pesquisa iremos treinar pelo menos mais dois profissionais experientes na rotulagem dos dados."

"O prazo para finalização do doutorado do pesquisador principal foi estendido para Set/2025. O pesquisador responsável foi contemplado com uma bolsa PrInt Capes para Doutorado Sanduiche. Local: Universidade de Surrey, Reino Unido. Data: 01/01/2024 a 30/06/2024. Processo: 88887.915510/2023-00."

Considerações sobre os Termos de apresentação obrigatória:
Trata-se de Relatório Parcial da Pesquisa.

Conclusões ou Pendências e Lista de Inadequações:
Para possibilitar êxito da pesquisa considero as justificativas de prorrogação aceitáveis.

Considerações Finais a critério do CEP:
Ressalta-se que cabe ao pesquisador responsável encaminhar os relatórios parciais e final da pesquisa, por meio da Plataforma Brasil, via notificação do tipo "relatório" para que sejam devidamente apreciados no CEP, conforme Norma Operacional CNS nº 001/13, item XI.2.d.
Qualquer alteração no projeto original deve ser apresentada "EMENDA",por meio da Plataforma Brasil, de forma objetiva e com justificativas para nova apreciação (Norma Operacional

001/2013 ¿ letra H).

Este parecer foi elaborado baseado nos documentos abaixo relacionados:

Tipo Documento	Arquivo	Postagem	Autor	Situação
Envio de Relatório Parcial	RelatoriosParcial_AIandPlaque.pdf	13/05/2024 09:22:45	Camila Lindoni Azevedo	Postado

Situação do Parecer:
Aprovado

Necessita Apreciação da CONEP:
Não

SAO PAULO, 04 de Junho de 2024

Assinado por:
Margareth Oda
(Coordenador(a))

Endereço: Av Prof Lineu Prestes 2227 - 1° andar , sala 02 - Administração
Bairro: Cidade Universitária **CEP:** 05.508-900
UF: SP **Município:** SAO PAULO
Telefone: (11)3091-7960 **Fax:** (11)3091-7960 **E-mail:** cepfo@usp.br
